# Carbon paste based sensor for sensitive Cr(III) ion determination in different water samples and anti-diabetic supplement

**DOI:** 10.1038/s41598-024-69176-y

**Published:** 2024-08-17

**Authors:** Aya E. Ali, Asmaa M. Mohamed, Gehad G. Mohamed

**Affiliations:** 1 Chemistry Department, Faculty of Science, Cairo University, Giza, 12613 Egypt; 2 Nanoscience Department, Institute of Basic and Applied Sciences, Egypt-Japan University of Science and Technology, New Borg El Arab, Alexandria 21932 Egypt

**Keywords:** Carbon paste, Schiff base ionophore, SEM and EDX study, IR, Potentiometric titration, Chemistry, Nanoscience and technology

## Abstract

A modified carbon paste sensor based on *N*,*N*′-(((ethane-1,2-diylbis(oxy))bis(2,1-phenylene))bis(methanylylidene))bis(pyridine-2-amine; BPA Schiff base as Cr(III) selective carrier was fabricated and studied in this work. The proposed sensor homogenization and mechanism of action was studied by infra-red (IR) and scanning electron microscope (SEM) with energy dispersive X-ray (EDX) tools. The sensor covered 1.0 × 10^−7^–1.0 × 10^–1^ mol L^−1^ linear range and a detection limit of 7.22 × 10^–8^ mol L^−1^ for Cr(III) with 20.17 ± 0.13 mV decade^−1^ Nernstian slope. 5 s was the response time of the prepared sensor and it was reproducible and stable for 3 months. The working pH range was 3.3–6.0 and it also works well to determine Cr(III) ion in presence of water miscible solvents up to 12.5% content of the methanol and 17.5% of ethanol. The electrode’s selectivity was studied using separate and mixed solution methods for selectivity coefficients determination and the sensor showed good selectivity relative to a variety of metal ions (selectivity coefficients = 1.01 × 10^−5^–8.57 × 10^−3^). In addition, the practical analysis value of the sensor was demonstrated by measurement of Cr(III) quantitatively in mineral water, supplement and also as an indicator electrode in Cr(III) against EDTA potentiometric titration with good reproducibility (RSDs of 0.91–2.15%).

## Introduction

Chromium(III) ion is an essential element for human health. Many studies showed that the action of insulin involves Cr(III) ion as an active element and there is a strong association between chromium(III) ion deficiency, high blood insulin and cholesterol levels^1^. Many people such as the elderly and those under long periods of stress such as pregnancy, infection, physical trauma, and strenuous exercise are especially at Cr(III) deficiency risk resulting in impaired insulin function, inhibition of protein synthesis and energy production, cardiovascular dysfunctions and type 2 diabetes^2,3^. In addition, Cr(III) ion deficiency can be a cause of many hormonal disorders such as polycystic ovarian syndrome (PCOS)^4^. Currently, chromium(III) salts including chelates have demonstrated extensive applications in medicine especially in healthy blood sugar regulation, dietetics, and sport nutrition^3,5^. The reasonable dietary intake for chromium(III) is 50–200 μg/day in adults^6^. Cr(III) is one of the essential trace elements in multivitamin with multimineral pharmaceutical formulations that contain only Cr(III) either in the form of chromium chloride (inorganic source) or organic source^7^. On the other hand, excessive Cr(III) ion exposure is harmful and the pollution by chromium ions is of considerable concern among various heavy metals as it has been used in many industries such as alloys manufacturing, chrome plating, industrial pigments, catalysts, leather tanning and wood treatment^7,8^, so the detection of Cr(III) that exist in many pharmaceutical preparations and polluted water is of great importance.

In spite of the presence of many high-level analytical techniques such as atomic absorption spectroscopy (AAS)^9,10^, inductively coupled plasma mass spectrometry (ICP-MS)^11^, fluorimetry^12^, high performance liquid chromatography (HPLC)^13^ and isotope dilution mass spectrometry^14^ that have been utilized for trace Cr(III) level determination. These methods have disadvantages in terms of cost, samples pretreatment and unsuitability for routine analysis in spite of their good sensitivities. They are also sophisticated instruments that need high quality technicians.

Many studies on the design and synthesis of sensitive and selective ionophores for ion-selective electrodes (ISEs) have been reported for various metal ions. However, there are only a limited number of reported studies on the development of highly selective ionophores for Cr(III) ion^7^ and from the point of view of the vital importance of Cr(III) in many pharmaceutical, biological and industrial samples, it is a challenging task to develop a new selective carbon paste electrode (CPE) for Cr(III) ion with low detection limit.

Carbon paste electrodes (CPEs) belong to promising electrochemical sensors of wide applicability. Baldwin described a simple method of direct mixing of a solid modifier to the paste^15^, which was the commencement of explosive research activity in this field. Presently, CPEs represented one of the most frequent types of working electrodes. The overwhelming number of CPEs, that were used worldwide, belong to pastes with insulating liquids (paraffin oil, silicon oil, bromonaphthalene, tricresyl phosphate and others). The basic requirements for a pasting liquid are their practical insolubility in the solution under measurement, low vapor pressure to ensure both mechanical stability and long lifetime, and their electrochemical inactivity in the potential window of interest case of voltammetric and amperometric applications. In contrast to the relatively complicated modifications of solid substrates, carbon pastes can be modified simply to obtain quantitatively new sensors with desired, often predefined properties^16–20^. The modified carbon paste must be held in special bodies. A holder for carbon pastes can be realized as a well drilled into a short Teflon rod^21^, a glass tube^22^, or a polyethylene syringe^23^ filled with the paste which is electrically contacted via a conducting wire. Such constructions are very simple and have advantage of renewable surface^24^.

Because they contain electron donor atoms like O, P, N, and S that can form coordination bonds with the toxic heavy metal ions, carbon paste electrodes modified with organic ligands have found widespread use in the electrochemical sensing of heavy metals^25^. Schiff’s base compounds are able to make selective and stable complexes with metal ions of compatible dimensions and can potentially be used in their determination and separation^26–28^. So, they have been extensively applied as ionophores for sensors construction for metal ions^29,30^. The chemistry of Schiff bases was a field of growing attention that has gained dominant importance due to their flexibility, straight synthesis, electron donating criteria, multidentate nature, and great complex formation constants for f- and d-block metals. Schiff bases are prepared by the condensation reaction between the –NH_2_ group and carbonyl group and this condensation reaction requires special conditions due to the Zwitter ion effect of amino acids. It was observed that pH played a crucial role in the condensation process^31^.

In this study, a newly synthesized Schiff base namely, *N*,*N*′-(((ethane-1,2-diylbis(oxy))bis(2,1-phenylene))bis(methanylylidene))bis(pyridine-2-amine; BPA) was applied as a selective and sensitive ionophore for Cr(III) ion in carbon paste matrix. The proportion of constituents of CPE was optimized and influence of pH, temperature and solvent type was studied. The optimized CPE was applied successfully for Cr(III) ion determination in real water and pharmaceutical samples and the obtained data matched the data obtained by atomic absorption spectroscopy (AAS) and this made the approach seem promising for regular analytical applications.

## Experimental

### Materials

Analytical grade chemicals were used in this paper. Solutions were prepared from a stock solution of 0.1 mol L^−1^ Cr(III) that was prepared from a sufficient quantity of Cr(NO_3_)_3_.6H_2_O supplied from Sigma-Aldrich, in distilled water and buffered at pH = 4.1 using acetate buffer. The working solutions were prepared daily by suitable dilution of stock solution. All other solutions used in interference studies were prepared from analytical grade chloride salts purchased from El Nasr Company as well. *o*-Nitrophenyloctyl ether (*o*-NPOE) was supplied from Fluka, while dioctyl phthalate (DOP) and dibutyl phthalate (DBP) were supplied from BDH. 2-Fluorophenyl-2-nitrophenyl ether (FFNE), tricresyl phosphate (TCP) and graphite powder (synthetic 1–2 μm) were supplied from Sigma-Aldrich.

### Apparatus

The potential measurements were carried out using a digital Hanna pH/mV meter (model 8417). Silver-silver chloride double-junction reference electrode (HANNA, HI 5311) in conjugation with the prepared carbon paste electrodes under study was used. Jenway 3505 pH meter was used for pH measurements. Digital burette was used for the potentiometric titration of Cr(III). Automatic pipettes Socorex Swiss (50–200 µL and 200–1000 µL) were used to measure the very small volumes whereas glass micropipettes were used to measure the large volumes. For surface analysis, SEM Model Quanta 250 FEG (Field Emission Gun) attached with EDX Unit (Energy Dispersive X-ray Analyses), with accelerating voltage 30 K.V., magnification14x up to 1,000,000 and resolution for Gun.1n) was used at The Egyptian Mineral Resources Authority Central Laboratories Sector. The FT-IR spectra were measured on a Perkin-Elmer 1650 spectrometer (4000–400 cm^−1^) in potassium bromide pellets at the Microanalytical Center, Cairo University, Egypt. Contact angle analyzer of model T200 manufacture by Biolin Scientific under condition of sessile drop recipe, droplet distilled water volume 4 µm and measure time 10 s was used.

### Synthesis of the used ionophore (BPA)

**Figure 1:**
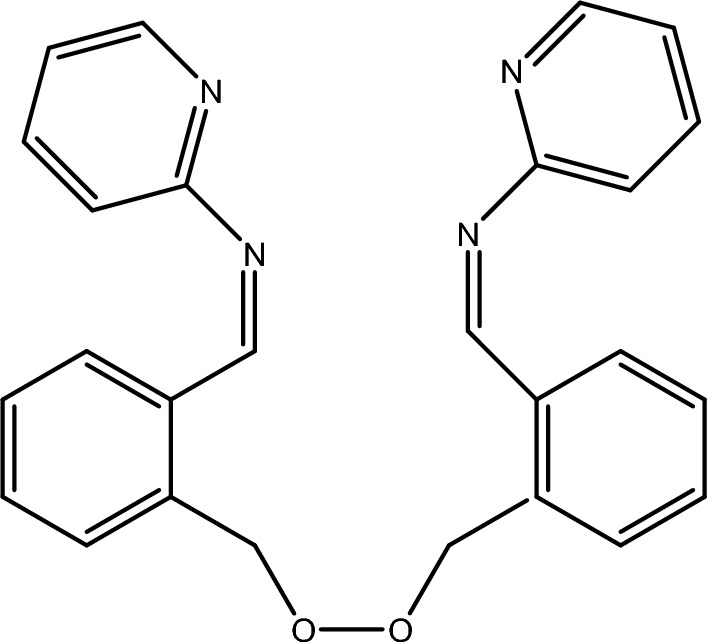
Structure of BPA ionophore.

2,2′-(Ethylenedioxy)bis(benzaldehyde) was prepared as previously described^32^. The bis-Schiff base was prepared by refluxing a mixture of 1.263 g 2-aminopyridine (13.446 mmol) and 1.60 g 2,2′-(ethylenedioxy)bis(benzaldehyde) (6.723 mmol) in ethanolic medium on a water bath for 3 h. The resulting yellow precipitate was filtered and purified by crystallization from ethanol, then dried in vacuum to give the desired bis-Schiff base ligand (Fig. 1). Yellowish brown solid’s yield was 94% and m.p. was 105 °C. Anal. calcd. for C_26_H_22_N_4_O_2_: C, 73.93; H, 5.21; N, 13.27; found: C, 73.76; H,5.09; N, 13.22. IR (KBr, ν cm^−1^): 1654 (C=N) pyridine, 1610 (HC=N) azomethine, 1015 (C–O–C)ether. ^1^H-NMR (DMSO-d_6_): d = 4.57 (m, 4H, OCH2), 7.06–7.68 (m, 16H, ArH), 8.20 (s, 2H, CH=N) ppm. ^13^C-NMR (DMSO-d_6_): 63 and 74 (CH_2_O), 159 and 160 (CH=N), 118, 119, 137, 145 and 156 (pyridine-C), 127–136 (Ar–C). Inhibition zone diameter (mm/mg sample): 15 (*Streptococcus pneumonia*), 14 (*Aspergillus fumigatus*).

### Synthesis and calibration of modified CPEs

First, the newly synthesized Schiff base *N*,*N*′-(((ethane-1,2-diylbis(oxy))bis(2,1phenylene))bis(methanylylidene)) bis(pyridine-2-amine) (BPA) was prepared to be applied as an ionophore. Then, the synthesized Schiff base ionophore was blended to the proper consistency in a mortar together with the liquefying substance and carbon powder, and the graphite paste was then placed in a Teflon container. Before use, the prepared electrode was conditioned in distilled water for 24 h^16–20,33,34^. To renew the CPE surface and remove any memory effects, the electrode was polished on a flat clean filter paper. A potentiometric cell made of an Cr(III)-CPE indicator electrode and a Ag/AgCl reference electrode was built for measuring electromotive force. Both electrodes were then placed in a beaker holding a Cr(III) solution that was adjusted to pH = 4.1 and connected to a milli-voltmeter. The proposed sensor was used to measure all electromotive forces utilizing the following assembly:

$$ {\text{Carbon}}\;{\text{ paste}}\;{\text{ electrode}}\left| {{\text{Cr}}\left( {{\text{III}}} \right) \, \;{\text{solution}}} \right|{\text{Ag}}/{\text{AgCl}}{-}{\text{KCl }}\;{\text{satd}}. $$

The recorded potentials were plotted as a function of −log [Cr(III)]. The resulting calibration graph was used for subsequent determination of unknown chromium ion concentration.

### Real samples examination

Actual water samples including river water (real water sample 1; the intake of Nekla station) and real water sample 2; the intake of Manshat El-Kanater network), were gathered and the pH of each sample was adjusted to a value of 4.0. Tap water samples used as matrix without any preliminary pre-treatment except the pH which was adjusted to 4.0 using HNO_3_ and/or NaOH. They were collected from Giza and Shoubra and received additions of Cr(III) ions in various concentrations (spiked water samples 1 and 2). The proposed modified CPE was used to analyze the chromitron supplement for insulin sensitization and diabetes prevention (35 mcg Chromium per capsule). To analyze chromium in chromitron supplement, the contents of one capsule were emptied and transferred into a silica crucible that was heated in a muffle furnace at about 650 °C for 7 h. Then, the obtained ash was dissolved in 20 mL HCl and diluted with distilled water in 100 mL measuring flask. The pH was adjusted at 4.0 using 0.1 mol L^−1^ NaOH and/or HNO_3_. The resulting solution was used for Cr(III) ion determination using the proposed sensor as an indicator electrode and applying standard addition method. The results were compared to those from atomic absorption spectroscopy (AAS).

## Results and discussion

### Optimization of carbon paste structure

**Figure 2:**
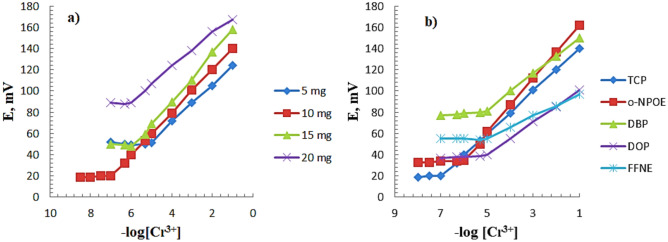
(**a**) Investigation of the effect of different ionophore content on the CPE’s response (electrodes No. 2, 3, 4 and 5, Table 1) and (**b**) Investigation of the effect of several plasticizers in the CPE at optimized conditions (electrodes No. 3, 6, 7, 8 and 9, Table 1).

Studies on the response characteristics of chromium(III)-ISEs based on *N*,*N*′-(((ethane-1,2-diylbis(oxy))bis(2,1-phenylene))bis(methanylylidene))bis(pyridine-2-amine) as an ionophore, graphite as conductive matrix and wide range of solvent mediators such as TCP, *o*-NPOE, DBP, DOP and FFNE were made to obtain the better response characteristics. Figure 2a and Table 1 showed that ionophore content of 10 mg has better behavior than the others with a good Nernstian response over a wide linear concentration range (1.0 × 10^−7^–1.0 × 10^−1^ mol L^−1^). By observing the results obtained in Table 1, the chromium ISE based on TCP exhibited a better Nernstian slope of 20.17 ± 0.13 mV decade^−1^ than *o*-NPOE, DBP, DOP and FFNE with slopes of 25.56 ± 0.74, 17.10 ± 0.84, 15.20 ± 1.07 and 10.40 ± 0.54 mV decade^−1^, respectively, as it is shown in Fig. 2b and Table 1. Therefore, TCP was chosen as plasticizer in the subsequent experiments. The proposed sensor was examined by different compositions and the effect of these paste compositions are given in Table 1. It is apparent from the table that the response of the electrode containing no ionophore (No. 1) has a sub-Nernstian slope of 9.08 mV decade^−1^ over a short range of concentration, while at the optimum composition of ionophore (10 mg) and applying TCP as plasticizer (electrode No. 3), the obtained slope was 20.10 mV decade^−1^ at the concentration range of 1.0 × 10^−7^–1.0 × 10^−1^ mol L^−1^. These results indicated the importance of existence of ionophore at the optimum amount. The applied ionophore in an ion-selective electrode (ISE) plays a very critical role in the selectivity and sensitivity of the synthesized electrode towards the target ion. The ion-partition between two immiscible phases and the affinity between the analyte and the ionophore form the basis of the potentiometric ion sensors mechanism^35^. The ionophore used in ISEs should have fast exchange kinetics and suitable formation constants as well as good solubility in the paste matrix and sufficient lipophilicity as not to lose the ionophore by leaching that can result in deteriorating the analytical signal over time^36^.

**Table 1:** Investigation of the optimum composition of the prepared CPE.

Electrode No	Composition of various components in carbon pastes (amount in mg)			Electrode characteristics		
	*N*,*N*′-(((ethane-1,2-diylbis(oxy))bis(2,1-phenylene))bis(methanylylidene))bis(pyridine-2-amine) ionophore, mg	Plasticizer (100 mg)	Graphite, mg	Slope ± SD*, mV decade^-1^	Linear range, mol L^-1^	Regression
1	0	TCP	250	9.08 ± 0.98	1 × 10^–4^–1 × 10^–2^	0.9881
2	5	TCP	250	17.90 ± 1.17	1 × 10^–5^–1 × 10^–1^	0.9979
**3**	**10**	**TCP**	**250**	**20.17 ± 0.13**	**1 × 10**^**–7**^**–1 × 10**^**–1**^	**0.9996**
4	15	TCP	250	22.36 ± 0.70	1 × 10^–6^–1 × 10^–1^	0.9977
5	20	TCP	250	15.88 ± 2.45	1 × 10^–6^–1 × 10^–1^	0.9961
6	10	*o*-NPOE	250	25.56 ± 0.74	1 × 10^–6^–1 × 10^–1^	0.9991
7	10	DBP	250	17.10 ± 0.84	1 × 10^–5^–1 × 10^–1^	0.9991
8	10	DOP	250	15.20 ± 1.07	1 × 10^–5^–1 × 10^–1^	0.9997
9	10	FFNE	250	10.40 ± 0.54	1 × 10^–5^–1 × 10^–1^	0.9989

*SD of 5 replicates.

Significant values are in bold.

### Selectivity study

Preliminary potentiometric response description based on CPE a wide variety of cations was obtained and the results are shown in Table 2. Between all these cations, the potential response of the proposed sensor towards the Cr(III) ion exhibited a better linear response characteristics with the concentration ranging from 1.0 × 10^−7^ to 1.0 × 10^–1^ mol L^−1^. Accordingly, paste components were optimized (Section III. 1) to give Nernstian response to chromium ion. The sensor with optimum composition showed a good Nernstian slope of 20.17 ± 0.13 mV decade^−1^ with a correlation coefficient of 0.9996 (n = 5).

**Table 2:** Potentiometric response of the proposed sensor against various cations and potentiometric selectivity coefficients for Cr(III) proposed sensor calculated by SSM, FIM and MPM.

Metal ions	Potentiometric response of proposed Cr(III) ISE towards various cations			Selectivity coefficients (K^Pot^_Cr(III), B_)		
	Slope ± SD*, mV decade^−1^	Linear range, mol L^−1^	R^2^	K^SSM^_Cr(III), B_	K^FIM^_Cr(III), B_	K^MPM^_Cr(III), B_
Ni(II)	14.25 ± 0.88	1 × 10^–4^–1 × 10^–2^	0.9785	6.25 × 10^–4^	1.02 × 10^–3^	4.10 × 10^–4^
Cd(II)	18.75 ± 0.78	1 × 10^–4^–1 × 10^–1^	0.9882	3.36 × 10^–3^	1.55 × 10^–3^	8.75 × 10^–4^
Co(II)	16.72 ± 0.78	1 × 10^–5^–1 × 10^–2^	0.9891	1.08 × 10^–3^	2.25 × 10^–3^	5.05 × 10^–4^
Cu(II)	20.45 ± 1.08	1 × 10^–5^–5 × 10^–2^	0.9896	8.27 × 10^–4^	1.45 × 10^–3^	6.83 × 10^–4^
Mn(II)	19.36 ± 0.97	1 × 10^–4^–5 × 10^–2^	0.9873	5.12 × 10^–4^	0.96 × 10^–3^	2.07 × 10^–4^
Zn(II)	11.98 ± 1.67	1 × 10^–4^–1 × 10^–1^	0.9880	7.25 × 10^–4^	2.66 × 10^–3^	6.24 × 10^–5^
Al(III)	10.85 ± 1.48	1 × 10^–5^–1 × 10^–2^	0.9894	1.01 × 10^–4^	4.15 × 10^–5^	2.03 × 10^–5^
Pb(II)	17.35 ± 1.06	1 × 10^–4^–5 × 10^–2^	0.9878	2.47 × 10^–3^	3.02 × 10^–3^	6.07 × 10^–4^
Fe(III)	8.47 ± 2.08	1 × 10^–5^–1 × 10^–2^	0.9895	4.07 × 10^–4^	7.88 × 10^–5^	5.13 × 10^–5^
Ce(III)	10.52 ± 2.08	1 × 10^–4^–1 × 10^–2^	0.9868	6.32 × 10^–4^	4.21 × 10^–5^	2.08 × 10^–5^
Ca(II)	6.05 ± 0.56	1 × 10^–4^–1 × 10^–2^	0.9887	1.06 × 10^–4^	1.85 × 10^–4^	1.00 × 10^–5^
Mg(II)	7.55 ± 0.82	1 × 10^–4^–1 × 10^–2^	0.9881	1.33 × 10^–4^	5.08 × 10^–4^	2.07 × 10^–5^
Ba(II)	9.74 ± 0.95	1 × 10^–4^–5 × 10^–2^	0.9879	8.74 × 10^–5^	2.55 × 10^–4^	1.22 × 10^–5^
Na(I)	25.09 ± 0.76	1 × 10^–5^–1 × 10^–3^	0.9867	6.01 × 10^–3^	8.44 × 10^–3^	1.05 × 10^–5^
K(I)	26.44 ± 0.93	1 × 10^–5^–5 × 10^–2^	0.9871	4.20 × 10^–3^	8.57 × 10^–3^	1.01 × 10^–5^

*SD of 5 replicates.

For studying the interference effect of metal ions other than Cr(III) ion on the CPE under study, selectivity coefficients (K_A,B_) were calculated according to three different methods. The first and second methods were separate solution method (SSM) and fixed interference method (FIM). SSM depends on measuring potentials of constant concentrations of primary Cr(III) ion and interfering ions, separately^37^. In this study, the concentration was kept at 1.0 × 10^–3^ mol L^−1^. On the other hand, the FIM depends on measuring potentiometric selectivity coefficient under mixed solution conditions. The emf of solutions, including varying activity of the primary Cr(III) ion and constant activity of the interfering ion a_B_ (1.0 × 10^–2^ mol L^−1^), was measured^20,38^. Both SSM and FIM are based on Nicolsky–Eisenmen equation^39^. Unfortunately, Nicolsky–Eisenmen equation suffers from limitations including non-Nernstian behaviour of interfering ions and inequality of the charges of primary and interfering ions^7,33^. So, a third method was applied in this study that is more recommended by IUPAC^39^ as it can get rid of these limitations and gives analytically relevant practical K_A,B_ values; this method is called matched potential method (MPM). According to the MPM, the selectivity coefficient is defined as the activity ratio of the primary ion (A) and the interfering ion (B) that gives the same potential change in a reference solution^38,41^. A solution of fixed activity of primary ion is used as a reference solution (a_A_) and the first change in potential upon changing the primary ion activity (a_A_') is measured, and then the interfering ion would be added to an identical reference solution until the same potential change is obtained. The ratio between the two activities of the primary ion A relative to the interfering ion B denotes the selectivity coefficient K^Pot^_A, B_ as shown in the following equation^42^:

$$ {\text{K}}_{{{\text{A}},{\text{B}}}} = \, \left( {{\text{a}}_{{\text{A}}} ^{\prime} \, {-}{\text{ a}}_{{\text{A}}} } \right) \, /{\text{ a}}_{{\text{B}}} $$

It should be noted that the concentration of Cr(III) used as primary ion in this study was 1.0 × 10^−5^ mol L^−1^. The resulting selectivity coefficient values obtained for the proposed Cr(III) sensor are given in Table 2. It is obvious that all cations would not significantly affect the selectivity of the present Cr(III) electrode and this was proved by three different methods of selectivity coefficient determination (SSM, FIM and MPM).The selectivity coefficients values were low if compared to the previous reported method^43^ indicating the better selectivity performance of this reported electrode.

According to the hard-soft acid base notion (HSAB)^44,45^, the “hardness” of an ionophore and metal ions determines how well they interact with each other. The coordination sites between ionophores and heavy metal ions are N, S, and O donating atoms and various atom binding sites such as O, N, S; N, O; N, S; O, S,…etc. N, O, and O, N are the hard binding sites in ionophores, whereas S and O, S are the soft binding sites. One may assume that the chelation between the Cr(III) ions and the nitrogen or nitrogen donor sites of the ionophores is the process by which the electrode sensed the Cr(III) ion.

### Water miscible solvent and pH effects on Cr(III)-MCPE performance

The electrode response was also investigated in water miscible organic solvents such as methanol and ethanol. This feature is important in cases that the analytical sample is not completely soluble in water, but it could be solved in partially water–alcohol mixture and hence the applied organic ionophore could be leached in such solvents, the applied ionophore stability towards organic solvents such as ethanol and methanol must be studied^7,20^. The results are given in Table 3. The potential of the electrode in 1.0 × 10^−3^ mol L^−1^ Cr(III) solution was found to be virtually constant up to 12.5% content of the methanol and 17.5% of ethanol which clearly suggested that proposed electrode can be applied to estimate the Cr(III) ions in presence of the water miscible organic solvents without any disturbance.

**Table 3:** Response of the electrode in binary alcohol-water system.

% of alcohol	Potential (mV vs. Ag/AgCl electrode)	
	Methanol	Ethanol
0	101	101
5	101	101
10	101	101
12.5	101	101
15	98	101
17.5	96	101
20	96	97
25	92	94

**Figure 3:**
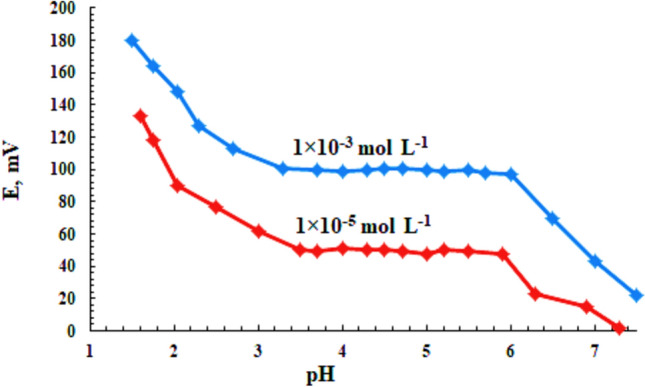
Influence of pH on proposed MCPE in different two Cr(III) concentrations in the pH range 1.5–7.5.

The pH influence on the carbon paste sensor was tested for 1.0 × 10^−3^ and 1.0 × 10^−5^ mol L^−1^ chromium(III) solutions over the pH range of 1.5–7.5 (using HCl or NaOH solutions for adjusting the pH) and the results are presented in Fig. 3. The potential responses remained pH independent over the ranges of 3.5–5.9 and 3.3–6.0 at concentration of 1.0 × 10^−5^ and 1.0 × 10^−3^ mol L^−1^ of Cr(III), respectively. These data were comparable to those previously reported^43^. The observed potential increase at lower pH values could be due to the response of the electrode to H_3_O^+^ ions. While, at higher pHs the formation of some hydroxyl complexes of Cr(III) ions may cause a decrease in potential responses^7,46–48^.

### Response time, detection limit, lifetime, repeatability and reproducibility

**Figure 4:**
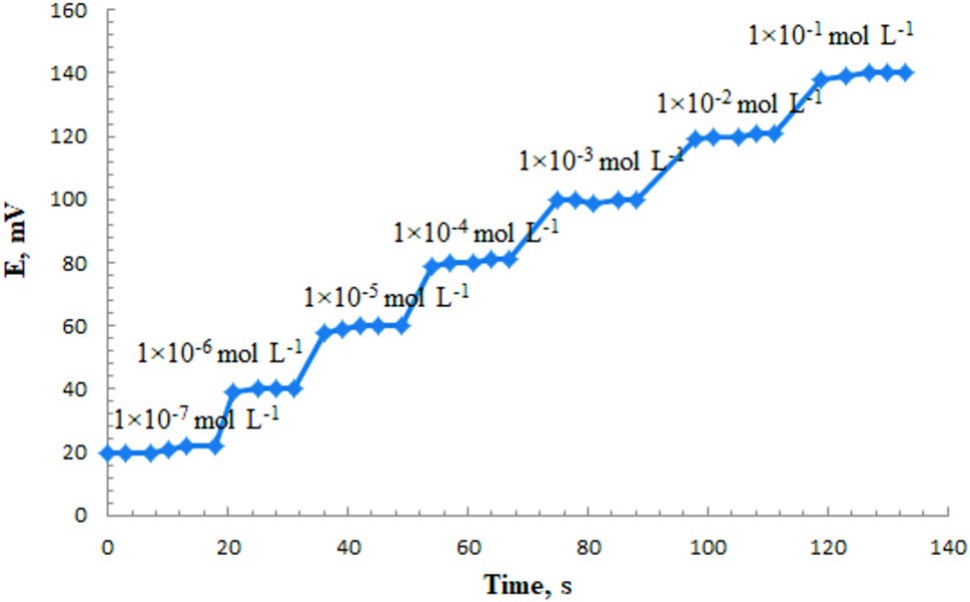
Dynamic response of the proposed sensor to step changes in Cr(III) concentrations.

According to IUPAC definition, the response time of the electrode was determined by measuring the time required to achieve a steady potential for a chromium solution ± 1 mV^7,49^. 5 s was the response time obtained for this MCPE with constancy up to 4 min (Fig. 4). This data was superior to the previously reported study which rescored 8 s response time^43^.

The limit of detection (LOD) as defined by IUPAC is the interpolated point at which the two linear segments of a calibration curve meet which are the Nernstian portion and the non-Nernstian portion of the calibration plot. Obviously to calculate LOD, the equations for the two linear regions are set equal to each other and the activity is solved for yielding LOD^7^. The limit of detection, which is evaluated according to IUPAC recommendations, was 7.22 × 10^−8^ mol L^−1^ Cr(III) ion. To investigate the electrode’s lifetime, calibration plots were obtained for 3 months (the sensor was heavily used for one hour each day) and the data showed that no expressive shift was detected for this period of time and the shelf life of the electrode can be extended by many months if it is kept in distilled water while it is not in use.

In the repeatability study, intra- and inter-days calibration curves of one electrode at five times were obtained, the calibration curves had been taken and the obtained average slope with its standard deviation was 20.17 ± 0.13 mV decade^−1^ (intra-day) and 19.73 ± 0.43 mV decade^−1^ (inter-day). The reproducibility was investigated by obtaining calibration curves of six similar electrodes at optimum paste composition then the slope of each electrode was determined and the average slope with standard deviation was 20.013 ± 0.56 mV decade^−1^.

### Electrode’s surface analysis and response mechanism

**Figure 5:**
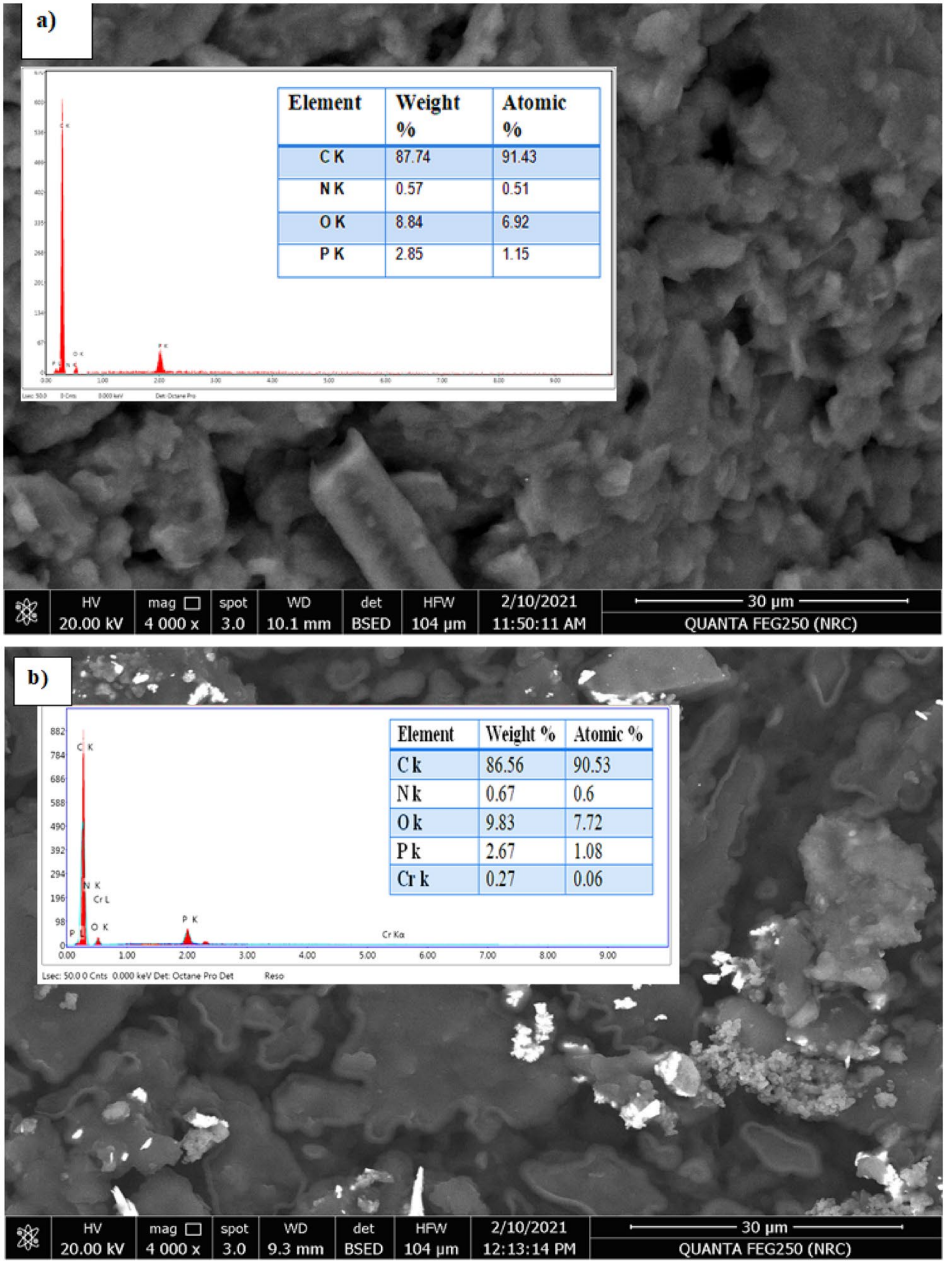
CPE surface SEM pictures before (**a**) and after (**b**) interaction with 1.0 × 10^–3^ mol L^−1^ Cr(III) ions with EDX analysis displaying the weight percentage of the various elements.

**Figure 6:**
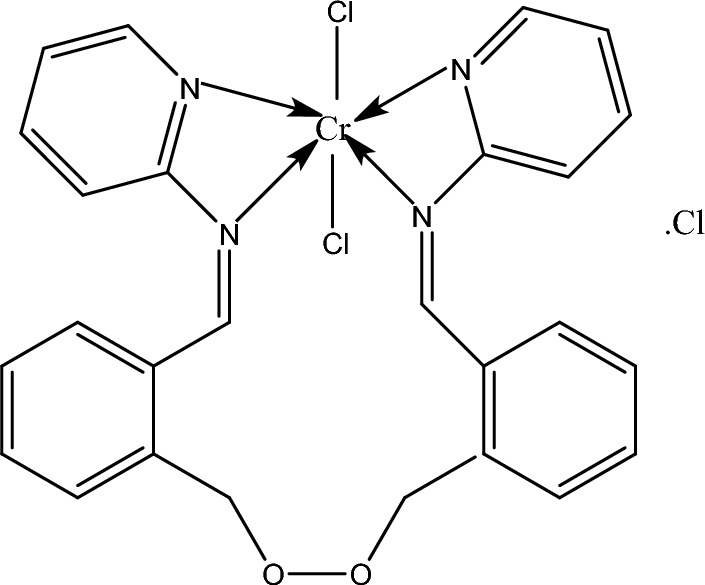
Suggested structure of the Cr(III)-BPA complex.

The applied ionophore played an important role in interacting with target metal ion by the aid of plasticizer which facilitated the extraction and entry of the target ion from water solution into the electrode’s surface. Herein our study a complexation mechanism was assumed as the type of interaction between the applied ionophore and target Cr(III) ions. Scanning electron microscope (SEM) and an energy dispersive X-ray analyzer (EDX) were the ideal tools for examining and proving this supposed mechanism on the graphitic paste surface. The surface morphology analysis can give crucial information on the homogeneity of the paste and can prove the entry and complexation of the target ions with the added complexing ionophore^16–20,34,46^. Figure 5 revealed the Cr(III) ion entry and interaction between target Cr(III) ions and the used ionophore and this was appeared as illuminated spots filling the graphite voids in SEM image (Fig. 5b) combined with EDX analysis which gave quantitative information about percentage of existing elements in the paste before and after soaking in 1.0 × 10^−3^ mol L^−1^ of Cr(III) ion for 1 h. As it can be shown in Fig. 5a and b, the Cr(III) percentage value of in the EDX study further proved the supposed interaction. In addition, these findings were corroborated by IR spectra of the paste before and after soaking in Cr(III) solution. Before soaking, the IR spectrum of the BPA ionophore showed the bands at 1653, 1612 and 1015 cm^−1^ which assigned to C=N of pyridine, HC=N azomethine and C–O–C etheric oxygen, respectively. After soaking, the bands of C=N of pyridine, HC=N azomethine and C–O–C etheric oxygen were found at 1655, 1610 and 1015 cm^−1^, respectively. The shift in the band position of the azomethine nitrogen and pyridine nitrogen can be accounted for their involvement in chelate formation with Cr(III) ion as shown in the suggested structure given in Fig. 6.

### Analytical application

Cr(III) ion concentration in real water samples and spiked water samples was found out applying direct calibration method. The obtained results are summarized in Table 4 It can be seen that in spite of the presence of other cations in these samples, the recovery % for the applied electrode was satisfactory and this can be attributed to the low detection limit and high selectivity of the synthesized Cr(III) sensor. In addition, the obtained data showed a very good correspondence with those obtained by AAS technique.

**Table 4:** Determination of Cr(III) in real and spiked water samples and chromitron supplement with comparison of the data with those obtained by AAS*.

Sample type	Taken, mg mL ^−1^	Found, mg mL^−1^		RSD% (n = 5)		Recovery %	
		Sensor calibration	AAS	Sensor calibration	AAS	Sensor calibration	AAS
Real water sample 1	–	4.58 × 10^–4^	4.61 × 10^–4^	1.85	1.93	–	–
Real water sample 2	–	2.08 × 10^–4^	2.11 × 10^–4^	2.03	1.97	–	–
Spiked water sample 1	5.20 × 10^–2^	4.98 × 10^–2^	5.00 × 10^–2^	1.67	1.55	95.77	96.15
	5.20 × 10^–4^	5.17 × 10^–4^	5.21 × 10^–4^	0.96	1.03	99.42	100.20
Spiked water sample 2	5.20 × 10^–2^	5.01 × 10^–2^	5.11 × 10^–2^	2.15	1.98	96.35	98.27
	5.20 × 10^–4^	5.23 × 10^–4^	5.21 × 10^–4^	1.06	0.98	100.58	100.19
Chromitron supplement	3.50 × 10^–4^	3.45 × 10^–4^	–	0.91	–	98.57	–

*At confidence level 95% (n = 5), F-test (tabulated) = 5.192 and t-test (tabulated) = 2.571. F-test (experimental) and t-test (experimental) values were (1.281–1.563) and (0.516–1.690), respectively.

The proposed electrode was as well applied successfully to quantify Cr(III) ion in chromitron supplement for insulin sensitization and diabetes prevention (35 mcg Chromium per capsule) using standard addition method (Table 4). The contents of one capsule were emptied and transferred into a silica crucible that was heated in a muffle furnace at about 650 °C for 7 h and the obtained ash was dissolved in 20 mL HCl and diluted with distilled water in 100 mL measuring flask. The pH was adjusted at 4.0 using 0.1 mol L^−1^ NaOH and/or HCl.

Finally, the proposed sensor was also used effectively as a working electrode in the potentiometric titration of 1.0 × 10^–3^ mol L^−1^ Cr(III) solution (25 mL) against 1.0 × 10^–2^ mol L^−1^ EDTA standard solution at pH = 4.5, the obtained recovery was 99.65% with RSD% = 1.026 (n = 5) and ΔE/ ΔV at end point was 180 mV mL^−1^ at 2.49 mL end point.

### Comparison with literature

It can be stated that this proposed MCPE for Cr(III) ion determination offered an increased selectivity and sensitivity in comparison with the latterly described potentiometric ISEs^7,34,43,46,50–55^ as shown in Table 5. This could be explained by the exceptional characteristics of the newly synthesized ionophore that could interact with Cr(III) ion specifically via complexation.

**Table 5:** Comparison of the proposed Cr(III)-MCPE with other reported Cr(III) ISEs.

References	Modifier	Detection limit, mol L^-1^	Linear range, mol L^-1^	pH range	Response time, s	Lifetime	Interfering ions with log k^pot^_Cr(III),B_ ≥ − 2
^7^	1-(2-(1H-Imidazole-1-yl)-1-(4-methoxyphenyl)ethylidene)-2-phenyl hydrazine	6.8 × 10^–8^	8.4 × 10^−8^–1.0 × 10^−2^	3.3–5.7	10	2 months	Fe(III), Al(III) and Ag(I)
^34^	1,3-Bis[4-amino-5-benzyl-1,2,4-triazol-3-ylsulfanyl]propane	8.0 × 10^−9^	1.0 × 10^−8^–5.0 × 10^−2^	2.3–5.2	10	3 months	No interference
^43^	Cr(III)-Schiff base ligand nano complex	1.0 × 10^–8^ (sensor I)	1.0 × 10^–8^ (sensor I)	2.0–6.0 (sensor I)	8 (sensor I)	98 days (sensor I)	Ce(III) and Fe(III)
		1.0 × 10^–9^ (sensor II)	1.0 × 10^–9^ (sensor II)	2.0–6.5 (sensor II)	5 (sensor II)	240 days (sensor II)	
^46^	Co-MOF	4.3 × 10^–9^	5.0 × 10^–9^–1.0 × 10^–2^	3.0–6.2	7	2 months	No interference
^50^	N-[4 (Dimethylamino)benzylidene]-6-nitro-1,3-benzothiazol-2-amine	2.0 × 10^−7^	4.0 × 10^−6^–1.0 × 10^−1^	2.8–5.1	15	5 months	No interference
^51^	4-dimethylaminobenzene	8.0 × 10^–7^	1.66 × 10^–6^–1.0 × 10^–2^	3.0–5.5	10	3 months	Ag(I), Fe(III), and Cu(II)
^52^	2H-1,4-benzothioazine-2,3(4H)dione dioxime	8.9 × 10^–7^	1.0 × 10^−6^–1.0 × 10^−1^	1.5–5.5	≤ 15	NM	Al(III), Ca(II), Ni(II) and Sn(II)
^53^	5-amino-1-phenyl-1H-pyrazole-4-carboxamide	5.3 × 10^−7^	1.0 × 10^−6^ − 1.0 × 10^−1^	3.2–6.3	10	2 months	Ca(II)
^54^	p-(4-acetanilidazo)calix[4]arene	NM*	9.8 × 10^−7^–1.0 × 10^−1^	2.8–5.7	10	12 weeks	Fe(III)
^55^	1-[(2-hydroxy ethyl) amino]-4-methyl-9H-thioxanthen-9-one	1.6 × 10^–7^	3.2 × 10^−7^–1.0 × 10^−1^	4.8–6.3	10	NM	Co(II), Sr(II), Mg(II), Ba(II) and K(I)
This study	*N*,*N*′-(((ethane-1,2-diylbis(oxy))bis(2,1-phenylene))bis(methanylylidene))bis(pyridine-2-amine)	7.22 × 10^−8^	1.0 × 10^−7^–1.0 × 10^−1^	3.3–6.0	5	3 months	No interference

*NM not mentioned.

It was clear from Table 5 that the proposed sensor has better LOD, wider linear concentration range and pH range comparable to the reported ones^50–55^. The most important point that makes the proposed sensor more applicable for determination of Cr(III) ion in real samples contaminated with di- and tri-valent cations is the advanced selectivity that results from the good affinity between the Cr(III) ion and the applied ionophore, fast exchange kinetics and suitable formation constants of the applied ionophore towards the target Cr(III) ion. It is clear from Table 5 that the proposed sensor doesn’t suffer from interference of other metal ions especially Fe(III), Al(III) and Ce(III) which makes this proposed MCPE superior to the other reported Cr(III) ISEs^7,43,51–55^. Potentiometric approach additionally offers advantages including simplicity, low cost, the lack of sample fabrication, and quick response and CPEs, especially, offer many advantages such as reliability, chemical inertness, affordability, renewability, and mechanical stability^16–20^. Moreover, the applied ionophore synthesis in this work offers simplicity and low cost than other reported Cr(III) sensors.

## Conclusion

In conclusion, the application of the fabricated carbon paste based solid contact electrode allows the sensitive and selective electrochemical detection of Cr(III) ion potentiometrically. The obtained results showed that the proposed electrode was applicable in wide concentration range with fast response time and long shelf time. The sensor can be successfully employed for the estimation of Cr(III) ion in real samples selectively even in the presence of other di- and tri-valent cations especially Fe(III), Al(III) and Ce(III) and this can be considered as a strength point of the proposed sensor over the other reported Cr(III) ISEs that suffered from other metal ions interference. This electrode in most cases is a good appendage to the previously reported Cr(III) selective electrodes. To the best of our knowledge, the use of carbon paste can improve sensitivity, stability, conductivity and renewability. Finally, this sensor is not to be used in aqueous media only, but it can also be applied for potentiometric analysis of Cr(III) ion in binary water–alcohol system.

## Data Availability

The datasets used and/or analyzed during the current study are available from the corresponding author on reasonable request.
